# Closing the Wearable Gap: Foot–ankle kinematic modeling via deep learning models based on a smart sock wearable

**DOI:** 10.1017/wtc.2023.3

**Published:** 2023-02-20

**Authors:** Samaneh Davarzani, David Saucier, Purva Talegaonkar, Erin Parker, Alana Turner, Carver Middleton, Will Carroll, John E. Ball, Ali Gurbuz, Harish Chander, Reuben F. Burch, Brian K. Smith, Adam Knight, Charles Freeman

**Affiliations:** 1 Department of Industrial and Systems Engineering, Mississippi State University, Mississippi State, MS, USA; 2 Human Factors and Athlete Engineering, Center for Advanced Vehicular Systems, Mississippi State University, Mississippi State, MS, USA; 3 Department of Electrical and Computer Engineering, Mississippi State University, Mississippi State, MS, USA; 4 Department of Kinesiology, Mississippi State University, Mississippi State, MS, USA; 5 School of Human Sciences, Mississippi State University, Mississippi State, MS, USA

**Keywords:** biomechanics, embedded electronics, performance characterization, sensors, soft wearable robotics

## Abstract

The development of wearable technology, which enables motion tracking analysis for human movement outside the laboratory, can improve awareness of personal health and performance. This study used a wearable smart sock prototype to track foot–ankle kinematics during gait movement. Multivariable linear regression and two deep learning models, including long short-term memory (LSTM) and convolutional neural networks, were trained to estimate the joint angles in sagittal and frontal planes measured by an optical motion capture system. Participant-specific models were established for ten healthy subjects walking on a treadmill. The prototype was tested at various walking speeds to assess its ability to track movements for multiple speeds and generalize models for estimating joint angles in sagittal and frontal planes. LSTM outperformed other models with lower mean absolute error (MAE), lower root mean squared error, and higher *R*-squared values. The average MAE score was less than 1.138° and 0.939° in sagittal and frontal planes, respectively, when training models for each speed and 2.15° and 1.14° when trained and evaluated for all speeds. These results indicate wearable smart socks to generalize foot–ankle kinematics over various walking speeds with relatively low error and could consequently be used to measure gait parameters without the need for a lab-constricted motion capture system.

## 1. Introduction

Many researchers have addressed the development of gait analysis systems, and many advancements have been made, but some challenges still need addressing. Gait movement is being increasingly investigated for two main goals: (a) monitoring and estimation of gait kinematics for movement tracking and addressing health-related concerns (Uddin et al., 2017) and (b) gait identification and recognition (Christ et al., 2013; Nair and Kendricks, 2016; Prakash et al., 2016; Triloka and Senanayake, 2017) for security purposes, identifying each person based on their unique movement pattern.

In conjunction with recent advances in deep neural networks, wearable device development provides the grounds to address the current gaps in gait analysis systems. This is the next study in our Closing the Wearable Gap (CWG) research (Luczak et al., 2018; Chander et al., 2019; Saucier et al., 2019a,b; Davarzani et al., 2020; Luczak et al., 2020b; Talegaonkar et al., 2020; Carroll et al., 2021; Turner et al., 2021) with the ultimate goal of designing a wearable solution “from the ground up” (Luczak et al., 2018) capable of accurately measuring kinematic and kinetic features of the foot and ankle during gait movement. The proposed study implements a wearable prototype designed by the research team, based on soft robotic sensors (SRS) embedded into a sock to track foot–ankle movement on a treadmill at varying speeds using deep neural networks.

The term that this publication series is based upon, “Closing the Wearable Gap” is derived from a series of interviews and conversations with strength and conditioning coaches at the professional and collegiate ranks across the continental United States (Luczak et al., 2020a). In 2018, when these interviews took place and this smart sock research project was discussed, this began its project life cycle via National Science Foundation (NSF) funding. The wearable technology space was expected to become a $15 billion market by 2021 (Market Reports Hub, 2015). In 2022, this market is now projected to be $264.5 billion by 2026, growing at a faster than expected pace (ReportLinker, 2021). Despite this value, the coaches interviewed identified that gaps exist between what wearables vendors are manufacturing and what human performance practitioners need; “Fools gold” was a term coined for wearable solutions by some of the most well-known strength and conditioning coaches based on their attempt to incorporate this technology into their training regimen (Luczak et al., 2020a). The lack of trust for the data from these devices based on their proprietary algorithms was viewed as a key issue (Luczak et al., 2018) and was further magnified by statements from the strength and training experts explaining that the data being collected wasnot the information they most needed to make better health and safety decisions for their athletes.

The common phrase used across two rounds of NSF I-Corps program interviews (Luczak et al., 2018, 2020a) was that the strength coaches needed “data collected from the ground up.” Because the lower body is a closed kinetic chain when in contact with the ground, all lower body joints are connected. What these strength and conditioning coaches were saying was that they wanted foot–ankle data because with it, they could then predict knee, hip, and lower back biomechanics. “From the ground up” was the focus of this team’s proposal to NSF for the design and productization of a wearable solution that would consistently and accurately collect foot–ankle joint movement and ground reaction forces through pressure. The term “Closing the Wearable Gap” is the goal of this team to make a wearable product that meet the needs requested by the health and safety practitioner, while this article series remains our detailed account of the studies and development processes needed to successfully build a wearable device that meets the needs of our eventual customer: the strength and conditioning coach. With a lack of transparency around product design and data collection of most wearable products, this series of papers continues to offer an in-depth, first-hand account of how a lab-accurate wearable can be designed and validated for outside-of-the-lab use.

While several previous research exists on ideal sensor placement for assessing gait (Boerema et al., 2014; Engineering and Teichmann, 2016; Mokhlespour Esfahani and Nussbaum, 2018), these papers predominantly use accelerometer-based sensors, which behave differently from the stretch sensors used in this project. With the linear relationship of change in capacitance or resistance with the stretch of the sensors, these sensors have already been validated against motion capture systems to efficiently capture joint kinematics, when placed across a joint axis (Luczak et al., 2018; Chander et al., 2019; Saucier et al., 2019a,b; Davarzani et al., 2020; Luczak et al., 2020b; Talegaonkar et al., 2020; Carroll et al., 2021; Turner et al., 2021). Hence, the positioning of these sensors was determined based on our previous research to accurately capture joint kinematics. In the Part II paper (Saucier et al., 2019b), authors investigated multiple placements to determine the most efficient and accurate placement for each sensor. Even though the lower extremity kinematics is seen as a closed kinematic chain, it is extremely common to analyze joint-by-joint kinematics. As such, analyzing ankle kinematics alone provides a greater depth of understanding of the body segment that serves as the interface between the body and the environment. Ankle and foot kinematics are extensively studied in various scenarios such as normal gait, running, during sporting events, during slips, trips, and falls and even in clinical population (MacWilliams et al., 2003; Monaghan et al., 2006; Jenkyn and Nicol, 2007; Dixon et al., 2012; Chander et al., 2015, 2017).

Some previous research studies have targeted developing sock prototypes with embedded sensors for the purpose of monitoring the kinetic and kinematics of the foot ankle. Preece et al. (2011) designed a sock prototype using a resistive strain sensor, which is knitted from a nonconductive elastomeric base material with a low modulus of elasticity. They defined two tasks to evaluate the performance of the prototype including identifying the three key gait events – heel lift (HL), TO, and HS – as well as estimation of ankle joint angle in the sagittal plane. Kinematic data were processed using static calibration to calculate ankle joint center and define segmental coordinate systems as well as to calculate foot angles using Cardan angles. The estimations achieved correlation coefficients of 0.78 and 0.56 for no shoe and shoed experiments of walking with participant’s self-selected speed. Previous research studies indicated contradictory results on the effect of walking speed on the lower limb kinematic features and the capability of sensors to track gait movement.

Mengüç et al. (2014) estimated gait angles of lower-body joints during walking and running with various speeds and argued that while the sensor variability remained low even during the run, their measured joint angles deviated with increasing walking speed. They suggested that this behavior indicates that the sensors would be more suitable for higher-level control of movement tracking as opposed to direct control over the absolute position of the joints. On the contrary, in another study (Gholami et al., 2019), the authors argue that convolutional neural networks (CNN) and sensor characteristics together are robust to change of speed and can track the foot ankle even with increasing the movement speed. Hanlon and Anderson (2006) performed a study to examine the effect of speed on the gait kinematic on various phases of gait movement and indicated that kinematic data is strongly influenced by speed. Researchers note an increase in accuracy when data used for comparison originates from the same speed range as predictions. They mention that speed depends on foot angle and gait phase; this means that altering the speed will change the peak and the pattern of gait movement curve.

CNNs and long short-term memory (LSTM) neural networks are two architectures frequently implemented by researchers to estimate joint angles and have provided promising results. Gholami et al. (2019) analyzed intra and interparticipant performance of CNN models using nine strain sensors for estimating joint angles in sagittal, frontal, and transverse planes during running with five different speeds: 8, 9, 10, 11, and 12 km/h. The proposed method achieved root mean squared error (RMSE) lower than 2.2° and 6.4°, and the *R*-squared was higher than 0.88 and 0.81, respectively, in intraparticipant and interparticipant scenarios for hip, knee, and ankle joints in sagittal, frontal, and transverse planes. However, the results indicate that the algorithm’s nonsagittal angle estimation is more challenging due to the lower range of motion in the transverse and frontal planes.

The presented study aims to track the foot–ankle movement using SRS and MOCAP systems while walking on a treadmill wearing the designed sock prototype and shoes. SRS and MOCAP measurements are compared together, and angle estimation models are developed by mapping SRS data into MOCAP signals. Linear regression, CNN, and LSTM models are employed to build intraparticipant models, and various training approaches have been designed to enhance the estimation of kinematic features. Moreover, to explore the effect of walking speed on the performance of SRS data and estimation models, trials with different walking speeds performed on a treadmill, and the generalizability of models over multiple speeds are examined. Finally, the estimation error of all models is compared, and reasons for possible inaccuracies are discussed.

### 1.1. New contributions to Closing the Wearable Gap research

The contributions of this study in comparison to previous studies conducted within Closing the Wearable Gap research are as follows:

- A new dataset of foot ankle kinematics of walking with multiple speeds and self-selected speed on a treadmill.
- Validation of a newly developed sock prototype during walking on a treadmill using two different deep learning methods.
- A step detection algorithm using pressure-based capacitive sensor located at the heel that uses an adaptive threshold.
- Assessment of longer walking trials in a gait study for wearable prototype validation.
- Further validation of the accuracy of stretch sensor technology compared to gold standard optical motion capture.

## 2. Material and methods

### 2.1. Participants

Ten healthy participants (four males: height, 170–178 cm; mass, 84–99 kg; foot size, 9.5–11 [US]; and six females: height, 157–175 cm; mass, 54–75 kg; foot size, 7–9.5 [US]) with no known gait impairments participated in the study. Participants were briefed about the experiment. Physical activity readiness Questionnaire (PAR-Q) (Shephard, 1988) and informed consent was obtained from the participants based on the approved protocol from the University’s Institutional Review Board) after fully explaining the protocol along with the risks and benefits (Protocol ID: IRB-19-502).

### 2.2. Data collection devices

The experimental procedure includes measurements of gait and dynamic ankle functions while walking on a treadmill using an SRS-based sock prototype, MOCAP, and GoPro recording systems. The study uses a new sock prototype developed by the MSU Athlete Engineering Team for estimating joint angles at the ankle joint complex. The sock comprises four stretch-based SRS and one pressure-based SRS, (StretchSense, Auckland, New Zealand), measuring four-foot movements – Plantarflexion (PFX), Dorsiflexion (DFX), Inversion (INV), and Eversion (EVR) – at the foot–ankle complex during static and dynamic movements. The pressure sensor is attached to the heel of the sock prototype to identify HS and automate gait cycle notation. This prototype is the most updated version, which is described in great detail in CWG P7 (Talegaonkar et al., 2020) and was used in CWG P9 (Carroll et al., 2021). Each sensor consists of an elastomer dielectric and elastomer electrode layer with silicon being used as the elastomer, which is then pressed onto a jersey knit backing. This backing allows for the sewing of the hook and eyes on the sensors so that they can be added or removed from the sock. The characteristic mechanism of the capacitance of the sensor is described in equation (1), where *ε*_0_ is the dielectric’s vacuum or permittivity of free space, *ε_r_* is the dieletric’s relative permittivity, *A* is the area of the overlapping electrodes, and *d* is the thickness of the dielectric layer (Keplinger et al., 2008; Huang et al., 2017). Measurement of the SRS was implemented using the StretchSense SPI sensing board (StretchSense, Auckland, New Zealand).
(1)

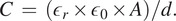

**Figure 1.**
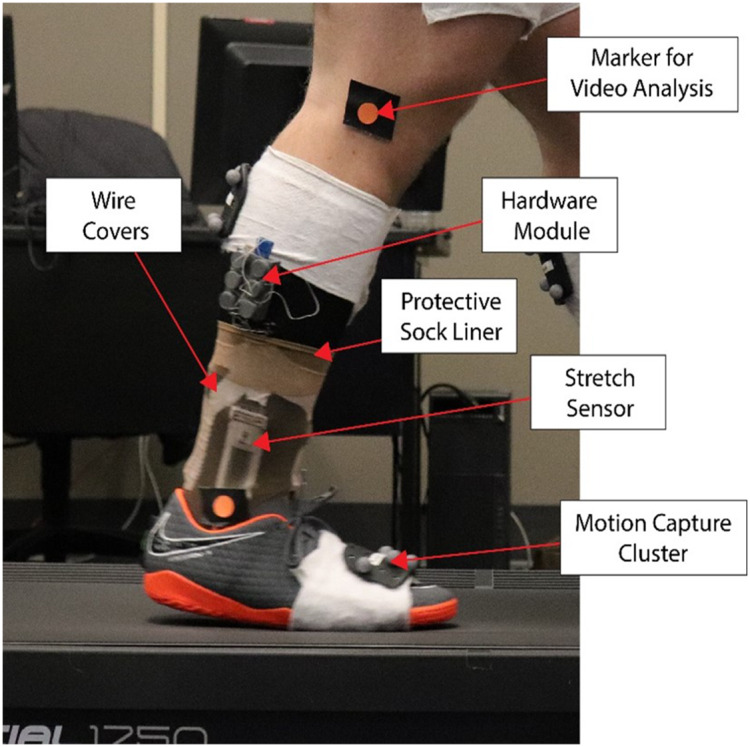
Participant right foot wearing designed sock prototype and shoes. Stretch sensor, and hardware module plus the protective sock liner, and wire covers are marked. MOCAP clusters mounted on the midfoot are also indicated. Two markers also are attached to the foot for GoPro video analysis.

This prototype uses an updated wire management system with individual wraps for each wire. In addition, a mesh sock was used to cover the smart sock while participants were wearing shoes, since the movement of putting on the shoe could snag the edges of sensors and cabling, damaging the prototype. The sensors are tightly conformed to the body using hook-and-eye mounts sewn to the sock. Two elastic bands sewn together are also wrapped around the foot and ankle to conform the substrate of the sensor to the sock, which are then further secured by the outer layer of the mesh sock. Figure 1 provides an illustration of the experiment setup with the sock prototype. Twelve Bonita 10 cameras were used as for the optical three-dimensional (3D) MOCAP system (Vicon, Oxford, UK), two GoPro cameras (placed to the lateral and rear of the participant), and a custom-developed SRS application was used to record the experiment data. GoPro cameras were used for review of the data after the data collection was completed.

### 2.3. Experimental setup and methodology

Height, weight, and shoe size were recorded for participant calibration into the MOCAP system. The participant was asked to don the smart sock embedded with the SRS and shoes. The researcher then placed seven retro-reflective marker clusters on the participant’s feet, on the middle third of each shank, the middle third of each thigh, and the lower back (L5S1). Joint centers were subsequently calibrated using an acrylic stylus (Innovative Sports Training, Inc., Chicago, IL) to create a relative 3D reference for the MOCAP system. The kinematic data were collected at a sampling rate of 125 and 250 Hz by SRS and MOCAP system, respectively. MOCAP system outputs two values, Flexion (FLX) and Inversion (INV). Flexion’s positive and negative values are related to SRS DFX and PFX, respectively, and positive and negative values of Inversion are related to SRS INV and EVR, respectively.

The treadmill (iFIT, NordicTrack, Logan, UT) walking experiment comprised of five trials. Prior to data collection, a 2-min warm-up session was conducted at 0.67 m/s for familiarization with the treadmill. The participant was instructed first to set their feet on the treadmill’s side rails to avoid tripping. The researcher would then set the treadmill speed to 0.67 m/s. Once the speed was set, the participant was asked to step on the treadmill carefully, keeping his or her arms to the sides and looking straight ahead, resembling normal walking. Once the participant walked for 2 min, the researcher stopped the treadmill, and the other researchers prepared the recording systems for the initial data collection. The MOCAP, MSU-built SRS application, and GoPros were utilized to record the gait data following this warm-up trial.

The researcher operating the MOCAP gave the command “3, 2, 1, GO,” wherein the researchers hit the record button on “2” for all three recording systems to manually sync the recording. One of the researchers also clapped to create a signal for audio synchronization of the two GoPro cameras. Next, the participant was instructed to get on the treadmill and briefly stand up on their toes before returning to normal standing, which served as a kinematic signal synchronization prompt for the MOCAP, GoPros, and SRS-app in time. The participant was then asked to set the treadmill speed at 0.67 m/s and walk for 3 min. After beginning this trial, the treadmill speed on the display was concealed so that the participant could not read it. At the end of this trial, recording devices were stopped, and the participant was asked to select a comfortable self-selected walking speed using the selection keys on the treadmill key control. Once a comfortable speed was selected, the researcher controlling the treadmill speed noted the participant’s self-selected speed, and the treadmill was paused briefly for a break.

**Figure 2.**
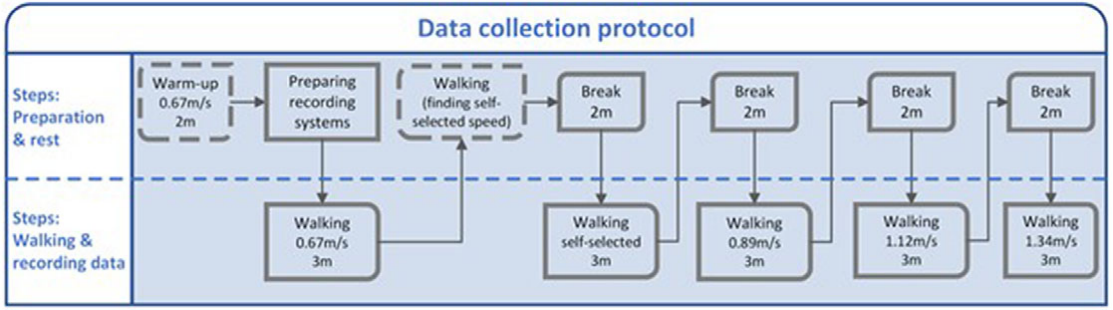
Data collection protocol. m and m/s stand for minute and meter per second respectively.

The participant was then asked to start the treadmill and walk at their self-selected speed for 3 min following the break. For the third, fourth, and fifth trials, the participant was asked to repeat the same synchronization procedure, and the trials were conducted at 0.89, 1.12, and 1.34 m/s, respectively. A rest break of 2 min was provided after each trial. Experiments were performed in the same order of speeds for all participants: 0.67 m/s, self-selected pace, 0.89, 1.12, and 1.34 m/s. This data collection protocol is summarized in Figure 2. Four walking speeds from 0.67 to 1.34 m/s (Boonstra et al., 1993; Meijer et al., 2009) were selected to include slow to fast walking speeds, as well as a self-selected speed. Participants walked for 3 min to gather enough gait cycles of stable walking while being adequate for training deep learning algorithms.

### 2.4. Data preprocessing

The difference in sampling frequency of the raw signals from the prototype and MOCAP system must be reconciled. SRS data were sampled at 125 Hz, while MOCAP data were sampled at 250 Hz. Next, SRS data were up-sampled using linear interpolation to match the MOCAP data rate. In the next step, SRS and MOCAP data were time-aligned using cross-correlation according to Rhudy (2014). For this purpose, the delay between SRS PFX sensor and MOCAP FLX signal has been calculated and data are shifted accordingly to mitigate the delay. The reason for considering PFX data for this purpose is that the participant standing on their toes at the beginning of each trial creates a high peak in the data acquired from this sensor to easily identify the time delay between the two systems. There are some trials where one of the measurement systems starts recording late and fails to record the timesteps when participant stand on their toes. These trials were aligned manually, when possible, otherwise they have been discarded from the dataset. The trials for participant 9 with 0.89 m/s and participant 10 with self-selected speed (0.94 m/s) are removed from the dataset due to this issue.

The last step of preprocessing is to remove gait cycles with noisy and outlier data. Outliers are removed only based on the MOCAP data. If the signals from the MOCAP system have outliers, the corresponding steps will be removed from both SRS and MOCAP data. For this purpose, we need to first annotate each gait cycle. The start of a gait cycle is marked according to the initial contact event (or HS) identified based on the heel pressure sensor data. A two-step algorithm has been designed for this purpose. The highest increase in the heel pressure sensor happens between HS and foot flat when the foot touches the ground. Therefore, in the first step, the algorithm finds the maxima of the first derivative of heel pressure sensor. Then in the next step, the algorithm searches the neighborhood of this timestep to find HS. In both steps, thresholds are implemented based on a specific percentile of timesteps to find the local maxima or HS.

After partitioning data, steps with outliers are identified and removed from dataset. First, the initial boundaries (IB) are determined based on all the gait cycles’ minimum and maximum values. The lower bound is the 0.25 quartile of the minimum values and the upper bound is equal to the 0.75 quartile of the maximum values. Then based on the following criteria the outlier steps are determined:

- MOCAP loses tracking in a few timesteps and creates lots of signal fluctuations: This type of noise is detected based on 1.5 IB.
- A few timesteps out of upper and lowers bounds determined based on two conditions: 10% of timesteps in one step are beyond the 3 IB or any timestep outside of 15 IB.
- The mean value of the signal is outside of 3 IB.

The cycles identified by each of these criteria are discarded from SRS and MOCAP datasets. A visual inspection of data indicated that the trial for participant 9 with 1.12 m/s right foot shifted upward at the first 20,000 timesteps. This trial (for the right foot) also has been removed from dataset.

Data from SRS and MOCAP then were smoothed using Savitzky–Golay (SG) filter to remove high-frequency noise without distorting the signal tendency. The window length and polynomial order of SG have been set to 31 and 5, respectively. New features have been generated and added to the input data based on SRS signals’ first and second derivatives using differential filtering in the time domain. SRS and MOCAP signals for each participant from all trials are normalized to have the mean of zero and standard deviation of one before feeding into the angle estimation models. Data from each trial has been partitioned into three parts, training data (first 60% of timesteps), validation (the following 20% of timesteps), and testing data (the last 20% of timesteps) before normalization. For the linear regression model there is no need for validation data and the training data consists of the first 80% of time steps. Normalization was performed based on the training data, and the same transformation has been applied on the validation and test data. Training data from each participant (all trials) was transformed so that have the mean of zero and the standard deviation of one.

### 2.5. Data analysis

This study implemented multivariate linear regression, LSTM, and CNN networks to model the relationship between SRS and MOCAP signals and estimate joint angles in sagittal and frontal planes. Models estimate FLX and INV signals of MOCAP system based on the DFX, PFX, INV, and EVR signals of the SRS system and their first and second derivatives. Data analysis was performed using Python 3.7. Regression models were trained using Sklearn library. All DL models are developed using TensorFlow 2.4.0. and Nvidia Cuda 11.0.3 on an Nvidia GTX 980 with i7-5960X CPU and 128 GB of RAM.

#### 2.5.1. Linear regression model

Linear regression develops a model to explain the linear relationship between one or more (multivariable) explanatory features and dependent variable. This method has been applied on gait movement data to analyze foot ankle kinematic features (Saucier et al., 2019a,b). A multivariate regression analysis is developed in this study to evaluate the linear relationship between the data from SRS and MOCAP signals. The least-squares approach was employed to fit the best-fitting line on the experiments’ observations and estimate the regression model’s coefficients.

#### 2.5.2. LSTM model

LSTM is a Recurrent Neural Network (RNN) architecture. RNN is a DL method designed and implemented for analyzing time-series data capable of capturing the sequential information hidden in time-series data. RNNs train the network based on gradient-based learning methods (Sra et al., 2012) and backpropagation (Chauvin and Rumelhart, 2013). This method has been successfully applied on many problems that deal with sequential data. However, when there is a long-term dependency among data, this method might encounter the vanishing or exploding gradient problem. LSTMs are designed to overcome the vanishing gradient problem by making modifications in cell design and operations. The long-term dependencies are encoded into the memory cell and three gates control its state – an input gate, output gait, and forget gait. By controlling the activations of forget gate and additive structure of cell operations, LSTM addresses the vanishing problem. More detailed explanations of RNN and LSTM networks are presented in (Sherstinsky, 2020).

Angle estimation models developed and trained using various recurrent network architectures are presented in this study. The network architecture consists of two to three LSTM layers, followed by a dropout layer to increase the model generalizability. Also, the effect of batch normalization has been evaluated by adding this layer in the architectures. At the end, one or two dense layers are added to find the nonlinear relationships between features and estimate the outputs. The number of units in LSTM and dense layers and the dropout rate (*p*) in dropout layers are tuned by trying 32, 64, and 128 units in LSTM layers with *p* equal to 0.2, 0.3, and 0.4. A learning schedule function has been designed to decrease the learning rate through the iterations. Models performed better with learning rate equal to 0.05 and decreased to 0.04. The model is trained for 40 epochs and a callback model checkpoint saved the best model based on the validation data to avoid overfitting on the training data. The Adam optimizer and mean absolute error (MAE) loss function were employed to train the network.

#### 2.5.3. CNN model

CNNs (LeCun et al., 1989 Lecun and Kavukcuoglu, 2010) that were originally designed to address the imagery data and automatically learn their spatial features from the data, are another type of neural network applied successfully on other applications including time series data analysis. The Convolutional (CONV), pooling, and fully connected (FC) layers are three main layers implemented in developing a CNN model. A CONV layer is the core building block of CNNs. In CONV layer a kernel convolves through an input layer to extract certain features. Various feature maps are extracted from the data by applying different kernels on a series of CONV layers. Then these features are mapped to the output through FC layers. Finally, pooling layers are applied on the feature maps and summarize the extracted features to reduce computational cost and increase the robustness of the model to noise and outlier (Mukhaimar et al., 2015).

Several CNN models are designed to find the best architecture on the gait data of this study. The baseline CNN model and parameters have been developed based on the network architecture introduced by Gholami et al. (2019) applied on the gait movement data for lower body kinematics estimation. Then the layers and parameters of the model have been tuned to achieve better performance on the dataset in the present study. Four to six CONV layers, and max pooling layers with the pool size of (2,1), which is applied only on the time dimension after some of CONV layers, have been implemented in building CNN models. GlorotUniform and GlorotNormal initializers, and Relu and LeakyRelu activation functions were employed in designing CONV layers. Then, a flatten layer followed by two FC layers to map these extracted features to the output features is added to the model. The number of filters varies between 50 and 256, and the kernel size was set to (3,1) and (5,1) to apply it on the time dimension to extract the sequential information of raw signals (Mukhaimar et al., 2015). Other parameters of models including learning rate, number of iterations, optimizer, and loss function follow the settings explained in LSTM models.

#### 2.5.4. Training strategies

To investigate the effect of varying walking speed in the performance of sensors, models have been trained and evaluated by three different strategies:

- Speed-specific: Models are trained by one specific walking speed, and the same walking speed as the test data to evaluate the performance of model.
- Multi-speed: Models are trained by all the walking speeds, and all the walking speeds are used as the test data to evaluate the performance of model.
- Speed-independent: Models are trained by all the walking speeds, and one specific walking speed is used as the test data to evaluate the performance of the model. This is similar to the previous approach in the training phase, except only data from one speed has been implemented to evaluate the performance.

**Figure 3.**
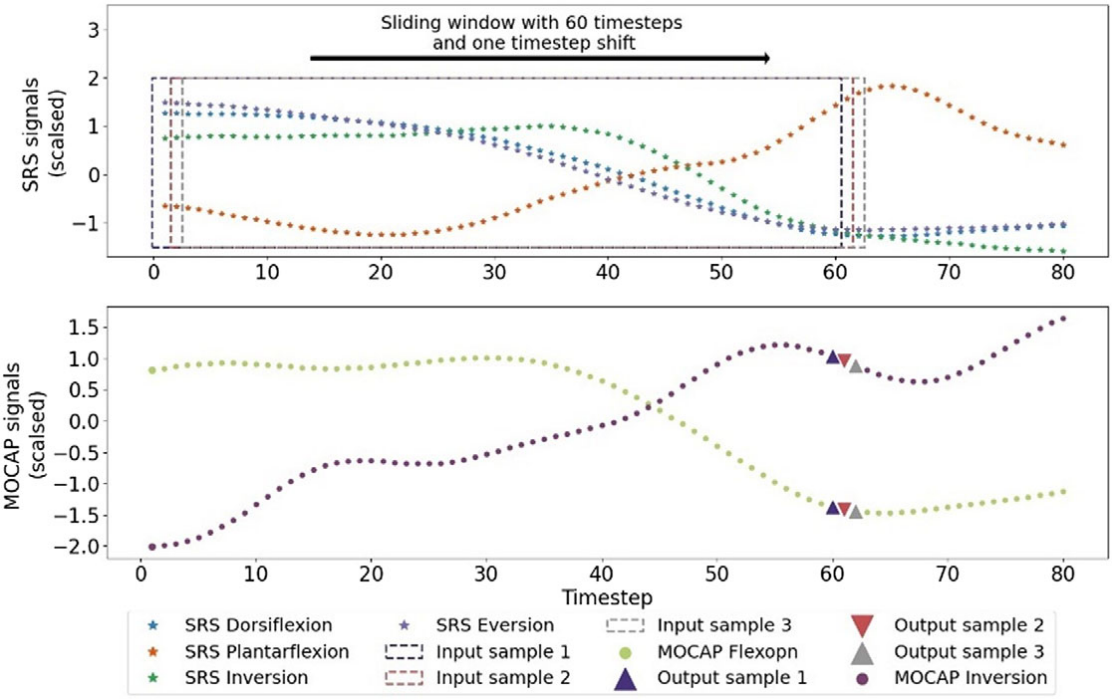
Preparing the sample frames of input and output data with sliding window. Only the SRS signals are plotted to keep the figure comprehensive. The number of timesteps in each sample (*N*) is set to 60. Top figure shows the SRS data and each rectangular indicated the timesteps in each sample. The corresponding output (MOCAP data) is plotted using triangles with the same color as rectangles.

A sliding window procedure has been employed to extract frames and shape the inputs for deep learning models. A sliding window with a length of *N* timesteps has been applied to each input signal and its derivatives to shape a frame of input data. The sliding window has been shifted by one timestep to extract the next frame. The shape of each input frame in the model is 12 × *N* and its corresponding output vector is the value of FLX and INV MOCAP signals at the timestep *N* with the shape of 2 × 1. This process is depicted in Figure 3. Only original SRS signals (not their derivatives) are shown on the plot for the sake of visual comprehension.

**Figure 4.**
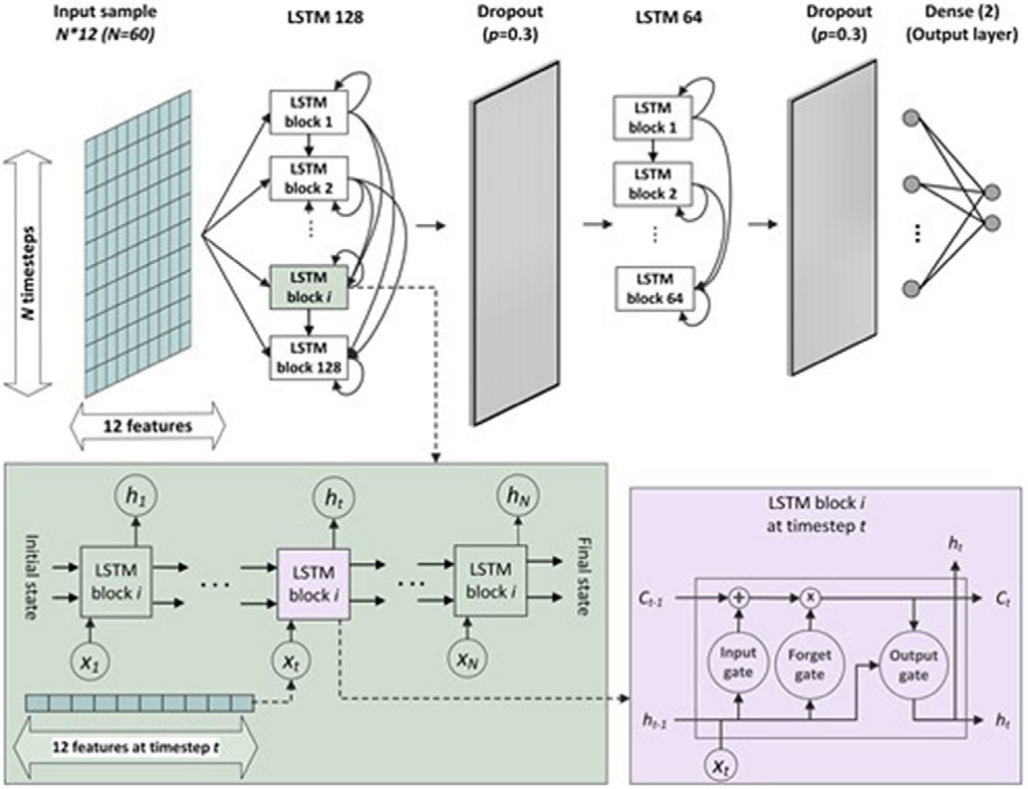
LSTM base model with 60 timesteps in each input frame.

The value of parameter *N* needs to be tuned for each model, and different values have been evaluated to find the optimal input shape. For this purpose, an initial network structure for LSTM and CNN has been set and evaluated using several values for *N* and the data from the left foot. The LSTM base model consists of two LSTM layers with 128 and 64 units each. The LSTM layer is followed by a dropout layer with a rate of 0.3, and there is a dense layer with the size of two to estimate two output values corresponding to FLX and INV signals of MOCAP system. The architecture of LSTM base model is presented in Figure 4.

**Figure 5.**
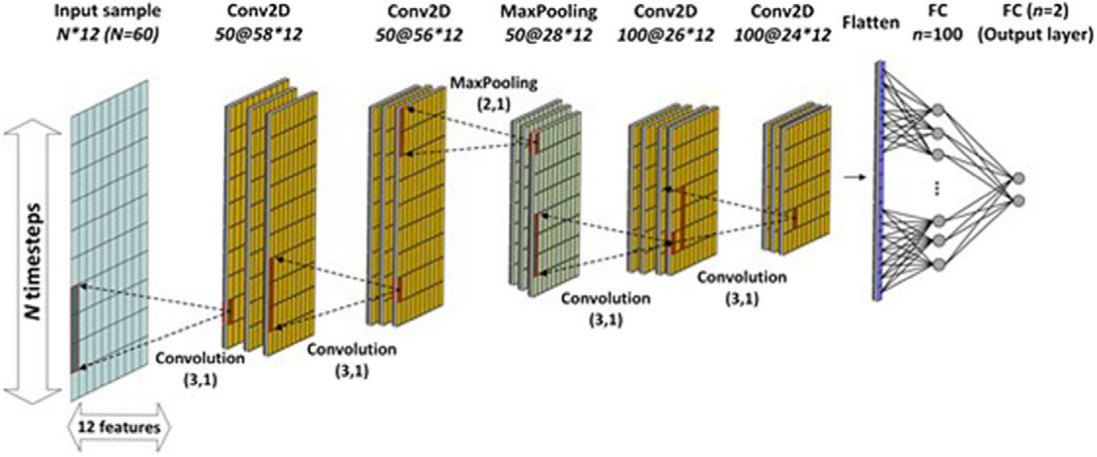
CNN base model with 60 timesteps in each input frame. Conv2D a@b*c: two-dimensional CONV layer with a, b timesteps, c features. FC = fully connected layer; Kernel = (3,1); pool size = (2, 1).

The CNN base model is based on the study of Gholami et al. (2019) and consists of two CONV layers with 50 filters in each layer followed by a MaxPooling layer. Then, there are two additional CONV layers each with 100 filters, a flatten layer, a dense layer with 100 units, and another dense layer with two units for estimation of outputs. Figure 5 shows the CNN base model’s architecture.

The results of the trained model have been evaluated based on three measurements, including MAE, RMSE, and *R*-squared. MAE and RMSE scores are in degrees matching the unit of MOCAP signals, and *R*-squared is a value between zero and one. Lower values in MAE and RMSE indicate lower error of estimation of the models and better performance. In contrast, higher values of *R*-squared are more desired as an indicator of better explanation of the variance in the MOCAP data.

## 3. Results

Table 1 indicates the average performance of CNN and LSTM models when trained and tested based on Speed-specific and Multi-speed approaches for various input sizes (*N*) to determine the optimal number of timesteps in each input frame. *N* has been set from 60 to 100 to divide the signals to the intervals between 120 and 400 ms (60–100 timesteps at 250 Hz). Comparing the results indicate that the *N* equal to 70 and 90 would provide better estimations for the CNN model when trained by each speed separately and by all the speeds, respectively. LSTM performs better with *N* equal to 90 timesteps regardless of the designing strategy.

**Table 1.** Average MAE, RMSE in degrees (°), and *R*-squared values for CNN and LSTM speed-specific and multi-speed models with different input sizes

			Average MAE (°)		Average RMSE (°)		Average *R*-squared	
Design strategy	Model	*N*	Flexion	Inversion	Flexion	Inversion	Flexion	Inversion
Speed-specific	CNN	60	1.635	1.131	2.295	1.464	0.877	0.807
		70	*1.449*	1.105	*2.023*	1.434	*0.902*	*0.824*
		80	1.987	1.195	2.739	1.536	0.812	0.754
		90	1.934	1.358	2.675	1.722	0.832	0.756
		100	1.636	*1.069*	2.333	*1.400*	0.873	0.802
	LSTM	60	0.959	0.739	1.396	0.988	0.969	0.916
		70	0.953	0.746	1.393	0.994	0.969	0.915
		80	0.950	0.739	1.384	0.984	0.970	0.916
		90	***0.944***	***0.734***	***1.377***	0.980	***0.970***	***0.918***
		100	0.963	***0.734***	1.398	***0.978***	0.969	***0.918***
Multi-speed	CNN	60	2.770	1.626	3.718	2.081	0.827	0.730
		80	2.866	*1.591*	3.825	*2.046*	0.811	*0.744*
		90	*2.632*	1.632	*3.578*	2.116	*0.837*	0.727
		100	3.674	1.959	4.946	2.537	0.656	0.553
	LSTM	60	2.258	1.270	3.082	1.661	0.878	0.831
		80	*2.232*	1.264	*3.061*	1.660	*0.885*	0.835
		90	2.239	*1.243*	3.064	*1.626*	0.881	*0.842*
		100	2.298	1.279	3.174	1.672	0.879	0.834

*Note:* The best *N* in each category is indicated in bold font, and the best performance score is in italics.

### 3.1. Multivariate regression analysis

**Figure 6.**
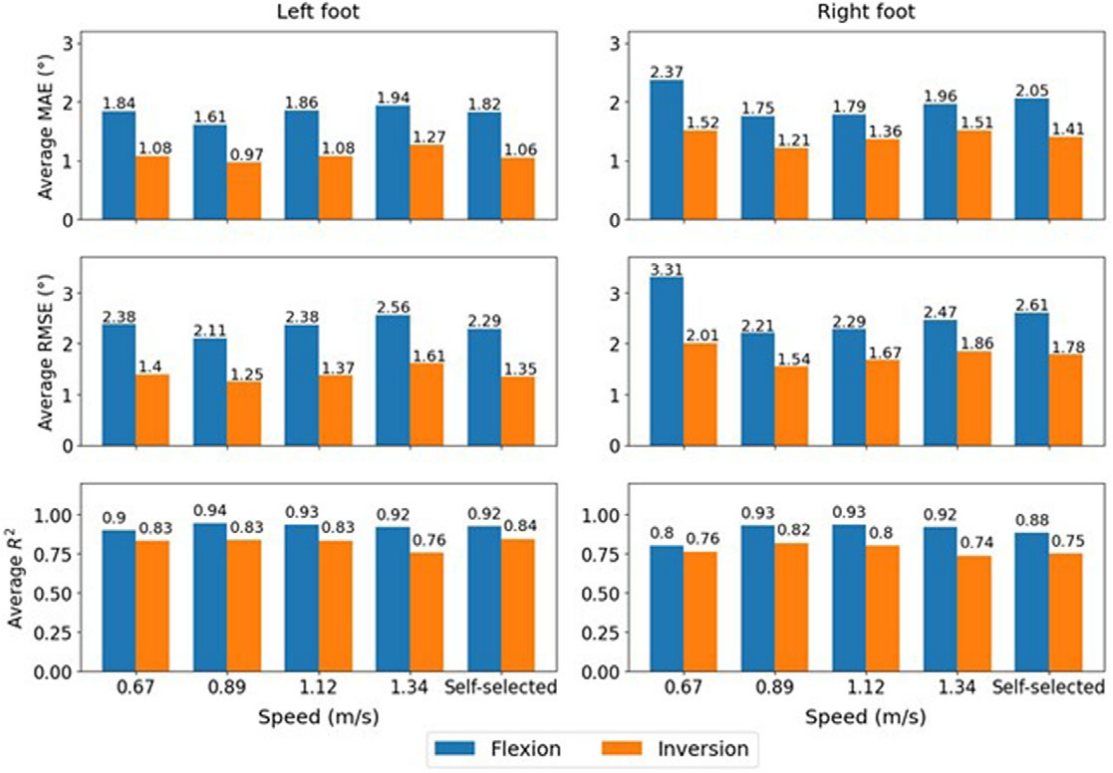
Average MAE (°), RMSE (°), and *R*-squared values for regression models for speed-specific design strategy.

Linear regression models were developed based on SRS capacitance and MOCAP joint angles for the left and right foot. This regression model finds the relationship between four SRS signals and their first and second derivatives (12 independent variables) with the independent variable that is FLX or INV signal of MOCAP system. The regression model is trained using the first 80% of timesteps in each trial, and the remaining data are used to evaluate the accuracy of regression models. The average of MAE, RMSE, and *R*-squared values are presented in Figure 6 for Speed-specific models.

### 3.2. Deep learning models

Several CNN and LSTM architectures have been developed and evaluated to better understand the network structure that fits the data. The number of layers and the units in each layer, kernel size, initializer, activation, and dropout rate are some of the model parameters that need to be tuned in each model. The input samples consist of 90 timesteps for all the models and design strategies, except for CNN with Speed-specific strategy, 70 timesteps. To limit the search space, the various network architectures have been evaluated using data from the left foot, and the best model architecture has been employed to train angle estimation model for the data from the right foot. The average MAE, RMSE, and *R*-squared results provided by the best architectures of CNN and LSTM are presented in Table 2. Train, validation, and test data consist of 60, 20, and 20% steps without shuffling. The detail of various network architectures and the score of performance measurements is presented in Tables A1 and A2.

**Table 2.** Angle estimation results of CNN and LSTM models for three design strategies

			Speed-specific		Multi-speed		Speed-independent	
Foot	Model		Flexion	Inversion	Flexion	Inversion	Flexion	Inversion
Left	CNN	MAE (°)	1.184	0.865	2.592	1.551	2.571	1.540
		RMSE (°)	1.663	1.153	3.490	2.008	3.094	1.881
		*R*-squared	0.959	0.89	0.839	0.743	0.825	0.643
Right		MAE (°)	1.366	1.034	2.035	1.647	2.000	1.624
		RMSE (°)	1.817	1.355	2.742	2.239	2.555	1.981
		*R*-squared	0.946	0.859	0.893	0.718	0.880	0.670
Left	LSTM	MAE (°)	0.944	0.734	2.140	1.211	2.125	1.201
		RMSE (°)	1.377	0.98	2.905	1.584	2.540	1.472
		*R*-squared	0.97	0.918	0.889	0.844	0.877	0.775
Right		MAE (°)	1.138	0.939	1.767	1.432	1.710	1.415
		RMSE (°)	1.558	1.238	2.492	1.991	2.239	1.754
		*R*-squared	0.957	0.875	0.912	0.772	0.903	0.730

**Figure 7.**
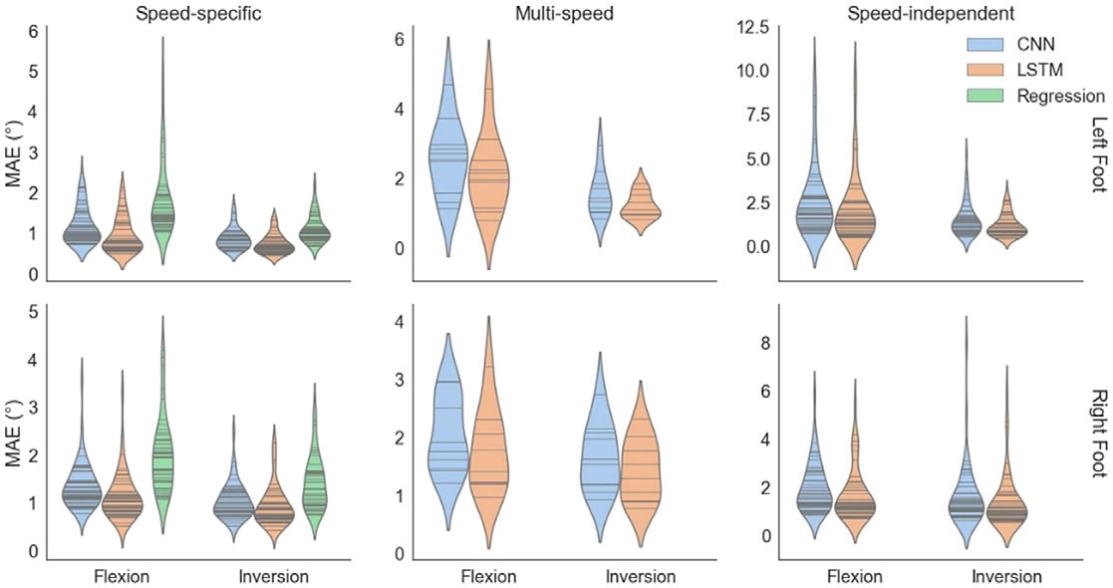
Violin plots of best trained model by regression, CNN, and LSTM for all three design strategies. Top figures are for the left foot and bottom figures for the right foot. Plots in the first column indicate the results of speed-specific models, plots in the second column correspond to the multi-speed models, and the last column of figures show the results of speed-independent models. The horizontal lines inside violin plots indicate MAE scores corresponding to each trained model (each trial in speed-specific, and speed-independent models, and each participant in multi-speed models).

The violin plots of MAE scores are presented in Figure 7. This graph shows kernel density distributions for different error values, the horizontal lines indicate the MAE score for each trial in Speed-specific, and Speed-independent models, and each participant in Multi-speed models.

**Figure 8.**
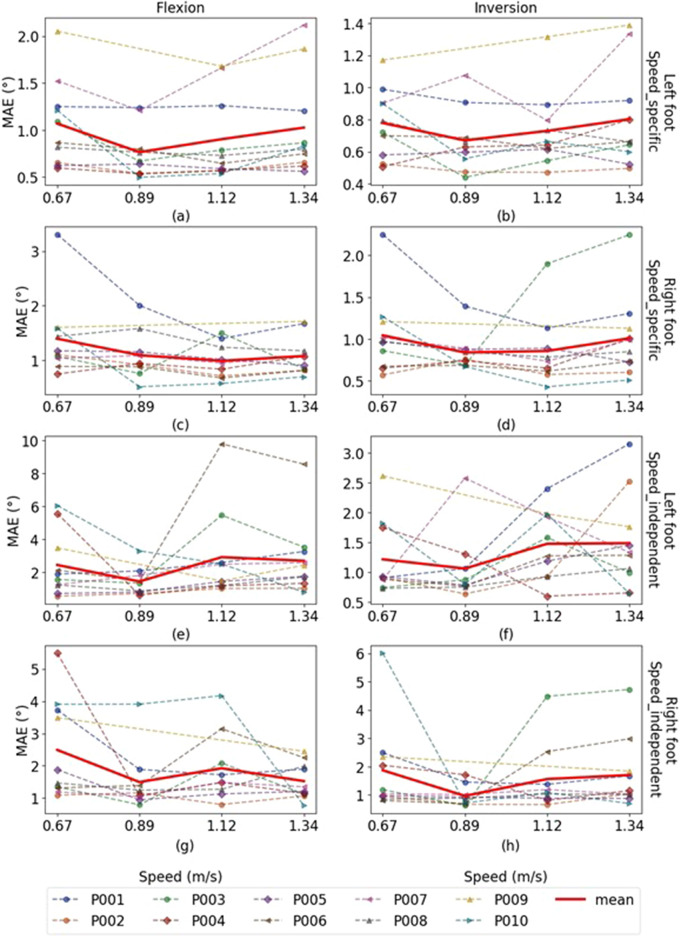
MAE scores trend lines over various walking speeds. The dotted lines indicate the MAE scores of angle estimations provided by LSTM models. The solid gray line in each figure is the average MAE scores over various participants. Part figures (a)–(d) are corresponding to speed-specific models and (e)–(h) for speed-independent models. The lower MAE scores are achieved by the speeds of 0.89–1.12 m/s.

Figure 8 illustrates the MAE scores for the FLX and INV angle estimations by the LSTM models to see the effect of speed change on the performance of SRS module and the generalizability of deep learning models over altering speeds. In each subfigure, MAE trend lines for each participant (dashed lines) and the average over various participants are presented.

**Figure 9.**
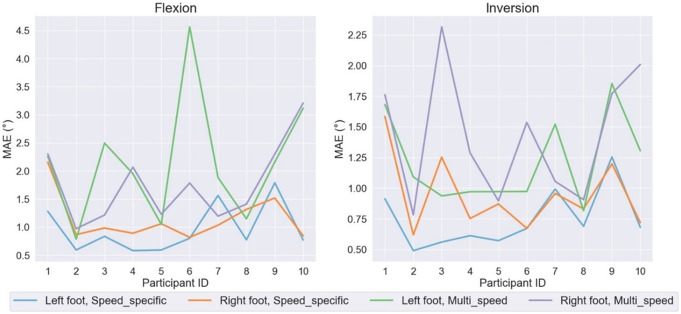
The average MAE scores for each participant on the FLX and INV joint angles.

Finally, the average MAE errors for each participant are presented in Figure 9 for Speed-specific and Multi-speed models. The MAE scores vary over different participants, and to some extend this could be explained by the issues explained above. Also, different gait cycle patterns while walking with various speeds account for the variation of MAE scores.

Several factors, including altering movement speed, contribute to determining how much a person’s gait pattern varies over different walking trials, which is an area for future work. The more severe estimation error for INV signal in participant 3, right foot with LSTM Multi-speed (Purple line in the right-hand side plot in Figure 9) initiates from different gait pattern of this participant over trials even with the same speed.

## 4. Discussion

Comparing the findings of this study with similar studies for the estimation of gait kinematics indicated that the performance of SRS and deep learning models are comparable to the literature. Gholami et al. (2019) designed similar experiments and achieved high *R* ^2^ values equal to 0.97 and 0.88 and RMSE values of 1.33° and 1.56° for the sagittal and frontal plane, respectively for the intraparticipant models. Gholami et al. (2019) also mentioned the performance of sensor and model remains consistent over various walking speeds, which is in agreement with the findings of the present study. Mengüç et al. (2014) used hyperelastic strain sensors based on microchannels of liquid metal embedded within elastomer and estimated ankle joint angle via linear regression. The authors set multiple walking speeds from 0.89 m/s (walking) to 2.7 m/s (running) and the RMSE values changed from 5° to 15°, indicating that increasing the walking speed reduced the accuracy of estimations. Sivakumar et al. (2021) developed a model for estimation of ankle joint angle using ground reaction forces measured from foot kinetic sensors and wavelet neural network with Gait Intervals feature selection (WNN)-GI and achieved RMSE of 2.772°± 1.070°.

In all three design strategies, LSTM achieved better performance followed by CNN model. This indicates that there is nonlinearity in the data that linear regression models could not successfully capture. The regression models trained by all the speeds were not able to follow the pattern of gait cycles and perform poor in estimating the peaks and are not reported. Generally, LSTMs are designed to process timeseries data while in contrast CNN is designed for analyzing visual imagery and exploiting the spatial structure of data, this could explain the superiority of LSTM as the gait data has time dependency over timesteps. Moreover, LSTMs capture the longer-time domain correlations while CNNs only generate local features depending on the kernel size.

Further investigation indicates that three main reasons for the higher error rate in models with lower performance include (a) noisy MOCAP data, (b) a shift in the readings of the MOCAP system, and (c) loss of tracking by MOCAP marker clusters. Regardless of the design strategy, angle estimation models are prone to errors when the signal is too noisy. Participant 1 with 0.67 m/s walking speed has the highest MAE value equal to 3.31° and 2.25°, respectively for FLX and INV signals in Speed-specific models. The row signals for this trial have been compared with data from participant 10, 1.34 m/s speed in Figure 9 to better illustrate this issue and its effect on the performance of angle estimation models. Reviewing the MOCAP recordings indicates that in some trials, marker clusters lost the movement tracking due to loose contact with the foot or getting covered up by the participant’s limbs or the treadmill. This issue could account for higher error rates in angle estimations.

Regarding the results in Figure 8, comparing the figures from the Speed-specific and Speed-independent models indicates a noticeable difference between the effect of speed in various designing strategies. In both design strategies, models for various speeds indicate similar range of performance scores and slightly better estimations corresponding to the speeds of 0.89–1.12 m/s walking speed. Also, looking at the data indicates that the average of participants’ self-selected speed is ≈ 0.89 m/s. Comparing plots in Figure 8b,f indicates same participant walking gait were more challenging for both design strategies (Participants 1,7, and 9) in INV estimation of the left foot. The very high MAE scores in plots from Figure 8e–h result from other issues mentioned before rather than the effect of speed and generalizability. Higher error rates in the plot for Figure 8e participant 6 with 1.12 m/s, participant 4 with 0.67 m/s are due to shifted MOCAP FLX signal, and participant 10 with 0.67 m/s is due to shifted SRS PFX signal. A shift in SRS INV signal accounts for high MAE corresponding to participant 1 with 1.34 m/s left foot (Figure 8f). A higher MAE score for participant 4 with 0.67 m/s in plot for Figure 8g is also a result of shifted MOCAP FLX signal. The plot in Figure 8h visualizes severe estimation errors for participant 6 with 2.5 and 1.34 m/s because of the shank sensors positioning, which are too high up and thus causing calibration issues and a shift MOCAP INV signal. Moreover, participant 10 with 0.67 m/s right foot is greater MAE scores due to noisy MOCAP signal and loss of track in the peaks of INV signal.

Foot ankle joint angles measured by MOCAP system were estimated based on data acquired by the MSU-designed prototype during walking on a treadmill using multivariate regression, LSTM, and CNN models. The MAE, RMSE, and *R*-squared were evaluated on these models to compare the SRS and MOCAP data and determine the best model for the angle estimation of foot ankle. LSTM model with lower MAE and RMSE scores and higher *R*-squared values outperformed the other methods, with two LSTM layers having 64 and 32 units, followed by a dropout layer with rate of 0.2 and one dense layer of size two at the end as the best LSTM model. Lower performance of regression models indicates the degree of nonlinearity in data and coupling between data from various sensors specially when all the trials train models with different speeds. This nonlinearity initiates from the variation in gait movement characteristics (even with same walking speed), altering walking speed, and variations in sensor readings.

Speed-specific models were trained to evaluate the performance of prototype and estimation models for various speeds. Results indicated that models perform uniformly over various walking speeds and a slightly lower estimation errors is achieved with a walking speed of 0.89 m/s, which is very close to the average of the self-selected speed of participants (≈0.89 m/s). Then, CNN and LSTM models were trained by data from all the trials performed by each participant with five different speeds, and results indicated their high performance that indicates the models can generalize over various walking speeds and estimate joint angles with relatively low error (Table 3).

**Table 3.** Summarization of study results compared to previous studies

Reference	Sensor type	Feature	Method (Metric)	Metric	Experiment	Remarks
Preece et al., 2011	Resistive strain sensor	Ankle joint angle (sagittal)	Correlation analysis	0.78 (no shoe), 0.56 (shoed)	Walking: Self-selected speed	Performance decreased in trial with shoe
Mengüç et al., 2014	Strain sensors microchannels of liquid metal	Joint angles lower body	Linear regression (RMSE)	5°–15°	Walking and running: Various speeds	Low Sensor variability even during the run Increasing the error with elevating walking speed
Gholami et al., 2019	Nine fiber strain sensors	Joint angles in sagittal, frontal, and transverse	CNN (RMSE)	2.2° (intraparticipant) and 6.4° (interparticipant)	Running: Five different speeds	Higher correlation with sagittal angles comparing to nonsagittal angles Similar accuracy at fast and slow speeds
Mundt et al., 2020	Inertial sensor	3D lower limb joint angle	LSTM (RMSE)	1.60 ± 0.57	Walking: Self-selected speed	Reduction of sensors using ANN Best Model with three sensors: pelvis, right and left shank
Conte Alcaraz et al., 2021	A single IMU	Lower body joint angles (sagittal plane)	GRNN, NARX, LSTM (RMSE)	2.57^∘^ (ankle) 2.12^∘^ (knee) 1.91^∘^ (hip)	Walking: Self-selected speed	LSTM has great potential in building digital twins for gait rehabilitation
Lu et al., 2022	8-channel sEMG signals	Lower limb multi-joint angles	CONV + LSTM (RMSE)	3.451 ± 0.29 (walk) and 3.891 ± 0.12 (Run)	Walk, run, stair descent, stair ascent, stand-to-sit, sit-to-stand, and jump locomotion	TDFD features for estimation of joint angles
Sivakumar et al., 2021	Foot kinetic sensors	Ankle, knee, and hip joint angles	WNN	2.86 (ankle) 3.98(knee) 2.07 (hip)	Walking: Self-selected speed barefoot	Using vGRF to reduces number of kinematics sensors
Presented work	SRS	Ankle joint angle (sagittal and frontal)	Linear regression, CNN, LSTM (RMSE)	Single speed: 1.46°, 1.11° All speeds: 2.69°, 1.78°	Walking: Five different speeds	Linear regression models could not successfully capture nonlinearity in data Models perform slightly lower with walking speed around self-selected

### 4.1. Future work

Estimation models in this study were developed for each participant, and the results indicate the designed prototype can measure the foot ankle data on various speeds and LSTM model is performing well on estimating joint angles in sagittal and frontal planes for each participant. The authors aim to establish interparticipant models based on the findings of this study and incorporate other demographic features as the future work. Lastly, the models presented herein could be compared to models generated from the GoPro footage collected during the study, to determine how well the SRS technology compares to other sensing modalities in predicting joint kinematic data.

### 4.2. Limitations

One of the limitations of this research is the sample size. With the novelty of the research and the development of the smart sock prototype, a smaller sample size that is representative of the general population was used. Additionally, previous analysis comparing these stretch sensors with motion capture systems have also used similar sample size, and hence this sample size was adopted based on previous research (Chander et al., 2019; Saucier et al., 2019b; Luczak et al., 2020b; Carroll et al., 2021).

Another limitation the authors faced during data collection was the quality of data collected by MOCAP system and its clusters. In addition, the gait cycles have been partitioned using a threshold-based algorithm and were controlled visually. Setting up force plates and employing a supervised algorithm for annotation of gait cycles will result in more accurate partitioning.

## Data Availability

Deidentified sensor data and software analysis conducted for this experiment will be available on the research team’s GitHub repository (https://github.com/msstate-athlete-engineering/soft-sensors-research). Standard operating procedures documents utilized for conducting the study will be included as well.

## Appendix A

CNN and LSTM estimation models and the layers of network and parameters and the score of performance measurements is presented in the Table A1 and Table A2. LSTM model performed better with the default initializer and Activation parameters; therefore, these two parameters are kept as default in LSTM models.

**Table A1.** Angle estimation results of CNN and LSTM models with Speed-Specific design on the left foot data

Model	Network		Kernel	Initializer	Activation		Flexion	Inversion
CNN	Conv(64)-MP-Conv(64)-MP-Conv(128)-MP-Conv(128)-MP-FL-D(128)-D(2)		(3,1)	GN	Relu	MAE (°)	1.207	0.9
						RMSE (°)	1.717	1.201
						*R*-squared	0.958	0.881
	Conv(64)-MP-Conv(64)-MP-Conv(128)-MP-Conv(128)-MP-Conv(256)-FL-D(128)-D(2)		(3,1)	GN	Relu	MAE (°)	1.654	1.154
						RMSE (°)	2.252	1.488
						*R*-squared	0.863	0.789
	Conv(64)-Conv(64)-MP-Conv(128)-Conv(128)-FL-D(128)-D(2)		(3,1)	GN	Relu	MAE (°)	2.094	1.294
						RMSE (°)	2.867	1.666
						*R*-squared	0.794	0.734
	Conv(64)-MP-Conv(64)-MP-Conv(128)-MP-Conv(128)-MP-FL-D(128)-D(2)		(5,1)	GN	Relu	MAE (°)	1.694	1.231
						RMSE (°)	2.319	1.58
						*R*-squared	0.846	0.771
	Conv(64)-Conv(64)-MP-Conv(128)-Conv(128)-FL-D(128)-D(2)		(3,1)	GN	Relu	MAE (°)	1.664	1.07
						RMSE (°)	2.327	1.399
						*R*-squared	0.878	0.808
	Conv(64)-Conv(64)-MP-Conv(128)- Conv(128)-FL-D(128)-D(2)		(3,1)	GN	Leaky Relu	MAE (°)	1.184	0.865
						RMSE (°)	1.663	1.153
						R-squared	0.959	0.89
	Conv(64)-MP-Conv(64)-MP-Conv(128)-MP-Conv(128)-MP-FL-D(128)-D(2)		(3,1)	GN	Leaky Relu	MAE (°)	1.181	0.878
						RMSE (°)	1.696	1.175
						*R*-squared	0.958	0.886
	Conv(50,3)-Conv(50,3)-MP-Conv(100,3)-Conv(100,3)-FL-D(100)-D(2)		(3,1)	GU	Relu	MAE (°)	1.449	1.105
						RMSE (°)	2.023	1.434
						R-squared	0.902	0.824
LSTM	LSTM(64)-Dr(0.3)-LSTM(32)-Dr(0.3)-D(2)		–	Def	Def	MAE (°)	0.955	0.737
						RMSE (°)	1.390	0.984
						*R*-squared	0.970	0.918
	LSTM(64)-BN-Dr(0.3)-LSTM(32)-BN-Dr(0.3)-D(16)-D(2)		–	Def	Def	MAE (°)	1.466	1.030
						RMSE (°)	2.015	1.368
						*R*-squared	0.942	0.854
	LSTM(128)-Dr(0.2)-LSTM(64)-Dr(0.2)-D(2)		–	Def	Def	MAE (°)	0.946	0.743
						RMSE (°)	1.377	0.992
						*R*-squared	0.970	0.915
	LSTM(128)-Dr(0.3)-LSTM(64)-Dr(0.3)-LSTM(32)-Dr(0.3)-D(2)		–	Def	Def	MAE (°)	1.007	0.763
						RMSE (°)	1.452	1.016
						*R*-squared	0.968	0.913
	LSTM(64)-Dr(0.3)-LSTM(32)-Dr(0.3)-D16-D(2)		–	Def	Def	MAE (°)	0.973	0.748
						RMSE (°)	1.409	0.994
						*R*-squared	0.969	0.917
	LSTM(128)-Dr(0.3)-LSTM(64)-Dr(0.3)-D(2)		–	Def	Def	MAE (°)	0.944	0.734
						RMSE (°)	1.377	0.980
						*R*-squared	0.970	0.918

*Abbreviations:* GN, GlorotNormal; GU, GlorotUniform; Conv, Convolutional layer; MP, Max pooling layer; FL, flatten layer; D, Dense layer; Dr, Dropout rate; BN, Batch Normalization; Def, Default values of LSTM network (Default Initializer, GlorotUniform; Default Activation, tanh). Best network architecture is highlighted in gray.

**Table A2.** Angle estimation results of CNN and LSTM models with Multi-speed and Speed-independent design strategies on the left foot data

					Multi_Speed		Speed-independent	
Model	Network	Initializer	Activation		Flexion	Inversion	Flexion	Inversion
CNN	Conv(50)-Conv(50)-MP-Conv(100,3)-Conv(100,3)-FL-D(100)-D(2)	GU	Relu	MAE (°)	2.632	1.632	2.623	1.605
				RMSE (°)	3.578	2.116	3.185	1.954
				*R*-squared	0.837	0.727	0.826	0.62
	Conv(64)-Conv(64)-MP-Conv(128)-Conv(128)-FL-D(128)-D(2)	GU	Relu	MAE (°)	2.592	1.551	2.571	1.540
				RMSE (°)	3.490	2.008	3.094	1.881
				*R*-squared	0.839	0.743	0.825	0.643
	Conv(64)-MP-Conv(64)-MP-Conv(128)-MP-Conv(128)-MP-FL-D(128)-D(2)	GN	Relu	MAE (°)	4.18	2.214	4.176	2.152
				RMSE (°)	5.499	2.826	5.238	2.641
				*R*-squared	0.589	0.513	0.556	0.373
	Conv(64)-Conv(64)-Conv(64)-MP-Conv(128)-Conv(128)-Conv(128)-FL-D(128)-D(2)	GN	Relu	MAE (°)	3.117	2.098	3.036	2.001
				RMSE (°)	4.177	2.681	3.716	2.438
				*R*-squared	0.737	0.597	0.735	0.486
	Conv(64)-Conv(64)-MP-Conv(128)-Conv(128)-FL-D(128)-D(2)	GN	Relu	MAE (°)	3.223	1.7	3.215	1.683
				RMSE (°)	4.365	2.231	3.95	2.093
				*R*-squared	0.715	0.664	0.697	0.523
	Conv(64)-Conv(64)-MP-Conv(128)-Conv(128)-FL-D(128)-D(2)	GN	Relu	MAE (°)	3.234	1.999	3.115	1.893
				RMSE (°)	4.436	2.661	3.87	2.338
				*R*-squared	0.7	0.599	0.708	0.503
	Conv(128)-Conv(128)-MP-Conv(256)-Conv(256)-FL-D(128)-D(2)	GN	Relu	MAE (°)	2.834	1.634	2.817	1.621
				RMSE (°)	3.811	2.128	3.373	1.985
				*R*-squared	0.811	0.716	0.796	0.612
	Conv(64)-Conv(64)-MP-Conv(128)-Conv(128)-FL-D(128)-D(2)	GN	Leaky Relu	MAE (°)	2.892	1.845	2.866	1.816
				RMSE (°)	4.431	2.85	3.843	2.462
				*R*-squared	0.651	0.551	0.658	0.443
LSTM	LSTM(128)-Dr(0.3)-LSTM(64)-Dr(0.3)-D(2)	Def	Def	MAE (°)	2.239	1.243	2.234	1.228
				RMSE (°)	3.064	1.626	2.677	1.516
				*R*-squared	0.881	0.842	0.869	0.779
	LSTM(64)-Dr(0.3)-LSTM(32)-Dr(0.3)-D(2)	Def	Def	MAE (°)	2.35	1.257	2.328	1.243
				RMSE (°)	3.236	1.662	2.811	1.541
				*R*-squared	0.861	0.837	0.849	0.773
	LSTM(64)-Dr(0.3)-LSTM(32)-Dr(0.3)-D(16)-D(2)	Def	Def	MAE (°)	2.439	1.306	2.411	1.287
				RMSE (°)	3.268	1.716	2.846	1.583
				*R*-squared	0.856	0.822	0.843	0.748
	LSTM(64)-BN-Dr(0.3)-LSTM(64)-BN-Dr(0.3)-LSTM(32)-BN-Dr(0.3)-D(2)	Def	Def	MAE (°)	3.035	1.749	3.022	1.730
				RMSE (°)	3.957	2.28	3.667	2.163
				*R*-squared	0.8	0.685	0.781	0.569
	LSTM(64)-Dr(0.2)-LSTM(32)-Dr(0.2)-D(2)	Def	Def	MAE (°)	2.140	1.211	2.125	1.201
				RMSE (°)	2.905	1.584	2.540	1.472
				*R*-squared	0.889	0.844	0.877	0.775
	LSTM(128)-Dr(0.4)-LSTM(64)-Dr(0.4)-D(2)	Def	Def	MAE (°)	2.291	1.271	2.273	1.251
				RMSE (°)	3.127	1.678	2.753	1.553
				*R*-squared	0.873	0.834	0.862	0.77

*Abbreviations:* GN, GlorotNormal; GU, GlorotUniform; Conv, Convolutional layer; MP, Max pooling layer; FL, flatten layer; D, Dense layer; Dr, Dropout rate; BN, Batch Normalization; Def, Default values of LSTM network (Default Initializer, GlorotUniform; Default Activation, tanh). Best network architecture is highlighted in gray.
